# Supplementary material for: Asynchronous magnetic resonance elastography: Shear wave speed reconstruction using noise correlation of incoherent waves
Source: Magn Reson Med. 2022 Oct 27;89(3):990–1001. doi: 10.1002/mrm.29502 (PMC9792433; doi:10.1002/mrm.29502)
Supplement: Supplementary file 1 — DATA S1 MATLAB code used to generate the numerical simulations of Figure 1 [file MRM-89-990-s002.zip › k-Wave/helpfiles/getBLI.html]

getBLI :: Functions (k-Wave)


# getBLI

Compute underlying Fourier band-limited interpolant (BLI).

## Syntax

```
BLI = getBLI(func)
BLI = getBLI(func, dx)
BLI = getBLI(func, dx, sampling_factor)
BLI = getBLI(func, dx, sampling_factor, plot_BLI)
BLI = getBLI(func, [], [], plot_BLI)
[x_fine, BLI] = getBLI(func, dx, sampling_factor, plot_BLI)
...
```

## Description

`getBLI` computes the underlying band-limited interpolant (BLI) of a discretely sampled 1D function assuming a Fourier basis. The example below shows the BLI of a discrete spatial delta function.

```
delta = zeros(20, 1);
delta(end/2) = 1;
getBLI(delta, 0.001, 10, true);
```

## Inputs

|  |  |
| --- | --- |
| `func` | 1D input function |

## Optional Inputs

|  |  |
| --- | --- |
| `dx` | spatial sampling [m] (default = 1) |
| `upsampling_factor` | spatial sampling used to sample the underlying BLI (default = 20) |
| `plot_BLI` | Boolean controlling whether the discrete input function and its BLI are displayed (default = false) |

## Outputs

|  |  |
| --- | --- |
| `BLI` | band-limited interpolant |
| `x_fine` | x-grid for BLI |

## See Also

`spect`
